# Ethanolic extract of *Caesalpinia bonduc* seeds triggers yeast metacaspase-dependent apoptotic pathway mediated by mitochondrial dysfunction through enhanced production of calcium and reactive oxygen species (ROS) in *Candida albicans*

**DOI:** 10.3389/fcimb.2022.970688

**Published:** 2022-08-24

**Authors:** Shan Sasidharan, Kumar S. Nishanth, Hareendran J. Nair

**Affiliations:** Department of R&D, Pankajakasthuri Herbal Research Foundation, Pankajakasthuri Ayurveda Medical College Campus, Trivandrum, India

**Keywords:** Apoptosis, *C. bonduc*, *C. albicans*, cytotoxicity, ROS, metacaspase

## Abstract

*Candida albicans* is a widespread disease-causing yeast affecting humankind, which leads to urinary tract, cutaneous and various lethal systemic infections. As this infection rate steadily increases, it is becoming a significant public health problem. Recently, *Caesalpinia bonduc* has received much attention from researchers due to its diverse pharmacological properties, including antimicrobial effects. Accordingly, we first planned to explore the *in-vitro* anticandidal potential of three extracts obtained from *C. bonduc* seeds against four *Candida* species. Initially, the anticandidal activity of the seed extracts was checked by the microdilution technique. Out of three seed extracts tested, ethanolic extract of *C. bonduc* seed (EECS) recorded the best activity against *C. albicans*. Hence, we next aimed to find out the anticandidal mechanism of EECS in *C. albicans*. The liquid chromatography quadrupole time-of-flight mass spectrometry (LC-QTOF-MS) analysis showed that the major compounds present in the EECS were tocopherols, fucosterol, linoleic acid, β-amyrin, β-sitosterol, campesterol, cassane furanoditerpene, Norcassane furanoditerpene and other diterpenes. To evaluate the cell death mechanism in *C. albicans*, a series of parameters related to apoptosis, viz., reactive oxygen species (ROS) production, membrane permeability, mitochondrial membrane potential, release of *cytochrome c*, DNA fragmentation, nuclear condensation, increased Ca^2+^ level in cytosolic and mitochondrial and activation of metacaspase, were analyzed. The results showed that EECS treatment resulted in the elevation of ROS, which leads to plasma membrane permeability* *in *C. albicans*. Annexin V staining further confirms the early stage of apoptosis through phosphatidylserine (PS) externalization. We further inspected the late apoptotic stage using DAPI and TUNEL staining assays. From the results, it can be concluded that EECS triggered mitochondrial dysfunction by releasing high levels of ROS, *cytochrome c* and Ca^2+^resulting in the activation of metacaspase mediated apoptosis, which is the central mechanism behind the cell death of *C. albicans*. Finally, a *Galleria mellonella*-*C. albicans* infection system was employed to assess the *in-vivo* potential of EECS. The outcomes displayed that the EECS considerably enhanced the recovery rate of *G. mellonella* larvae from infection after the treatment. Additionally, EECS also recorded low hemolytic activity. This study thus spotlights the anticandidal potential and mechanism of action of EECS against *C. albicans* and thus delivers a promising treatment approach to manage *C. albicans* infection in the future.

## Introduction

Urinary tract infections (UTIs) are predominately identified as contagions caused mainly by pathogenic microorganisms in hospital and community settings (Fisher, 2011; Dias, 2020). Pathogenic fungi and bacteria act as the major etiologic agents of UTIs (Dias, 2020). Scientific evidence indicates a reduction in the percentage of UTIs caused by pathogenic bacteria such as *Escherichia coli*, *Proteus* species and *Pseudomonas* species and a rise in the rate of UTIs caused by pathogenic fungi, specifically albicans and non-albicans *Candida *species in patients diagnosed with UTIs (Dias, 2020). *Candida albicans* is the predominant *Candida* species in patients affected by UTIs. Other non-albicans *Candida* species such as *C. glabrata*, *C. parapsilosis, and C. tropicalis* are also associated with UTIs (Dias, 2020). Moreover, the incidence of *Candida* species, particularly *C. albicans* in urine is considered as candiduria and its severity varies from asymptomatic candiduria to severe clinical sepsis (Gharaghani et al., 2018). Unfortunately, the global death rate of catheter-related candidemia has wangled more than 40% in recent years (Chen et al., 2019). Additionally, *C. albicans* can cause colonization on the catheter surface, followed by proliferation and biofilm development. Once the biofilm is formed, it is very tough to eradicate, which will make the condition much more severe. Presently, there are only three important classifies of drugs approved to treat and manage infections associated with* Candida* species. Moreover, the efficacy of the antifungal medications is highly compromised due to the development of various multi-drug resistant fungal strains, especially *Candida* species (Lee et al., 2021). Hence, it is necessitous to find novel antifungals to manage various pestilences caused by pathogenic *Candida* species. Natural antifungal agents, especially various novel phytochemicals from medicinal plants, may be a better alternative tactic to deny the increase of multidrug resistance in pathogenic fungi affecting humans.

At present, various scientists around the globe are concentrating on discovering novel medicinal plants to find natural antifungal compounds with less toxicity to manage the problems associated with drug resistance (Qu et al., 2019). *Caesalpinia bonduc* is a significant medicinal plant extensively spread throughout the tropical and subtropical regions of the world. Various parts of this plant have widespread use in the folk medicine system to treat a wide range of diseases affecting humans. Moreover, it is well reported that the seeds of this plant hold diverse pharmacological activities, including antibacterial and antifungal properties (Billah et al., 2013). The seeds of this plant are rich with alkaloids, glycosides, flavonoids, saponins, sterols, terpenoids, etc. (Kandasamy and Balasundaram, 2021). The pharmacological activity of the seeds is due to the occurrence of these bioactive phytochemicals in rich quantities. However, to our best knowledge, no investigations have been conducted to detail the anticandidal property of extracts obtained from *C. bonduc* seeds.

In this study, we intend to study the anticandidal potential of three *C. bonduc* seed extracts, followed by investigating the mechanism of action of EECS against *C. albicans *to demonstrate that EECS has the potential to develop as a new antifungal drug with significant therapeutic value from natural sources. To explicate the mechanism of action, we examined apoptotic effects triggered by EECS, which include the generation of ROS, its effect on membrane permeability followed by a rise in intracellular Ca^2+^ and mitochondrial dysfunction preceded by release of *cytochrome c*, PS externalization, nuclear condensation, DNA fragmentation/disintegration and metacaspase activation. From the experiments, we found that the treatment with EECS ends in apoptosis in *C. albicans* through the overproduction of ROS and Ca^2+^, which then leads to mitochondrial dysfunction leading to metacaspase-mediated apoptotic cell death. All these results suggest the potential of EECS to serve as a new natural antifungal medication against infections caused by *C. albicans* in the future.

## Materials and methods

### Collection of seeds

The *C. bonduc* seeds were collected from in and around Ernakulum (9.98°N 76.28°E), Kerala, India between January to April 2019. The seeds were authenticated and identified by Dr. Dan Mathew, a Senior Scientist at JNTBGRI, Trivandrum, Kerala, India. The specimens of the seeds were deposited in the JNTBGRI.

#### Preparation of *C. bonduc* seed extracts

Healthy *C. bonduc* seeds were selected for the extraction process. The seeds were washed in water and thoroughly dried at 45˚C using a hot air oven. After drying, the seeds were powered using a mixer grinder. The ethanolic, hydroalcoholic and aqueous extracts of the seeds were prepared separately by macerating 100 gm of powdered seeds in 0.5 L of absolute ethanol, water: alcohol (50:50) and distilled water for 72 h. After maceration, seeds materials were carefully removed by passing through a cheese cloth. The extraction procedure was repeated twice, and all collected filtrates were combined and separately dried with the assistance of a rotary vacuum evaporator at 40°C. The left-over solvents in the extracts were thoroughly removed using a vacuum oven. Finally, the extracts were subjected to freeze-drying using a lyophilizer. The three dried extracts [Ethanolic extract of *C. bonduc* seeds (EECS), Hydroalcoholic extract of *C. bonduc* seeds (HECS) & Aqueous extract of *C. bonduc* seeds (AECS)] thus obtained were stored at 4°C for future studies. (Sasidharan et al., 2022).

### Test *Candida* species and growth media

In this study, we have used four *Candida* species obtained from the Microbial Type Culture Collection Centre (MTCC), IMTECH located in Chandigarh, India. The *Candida* species used for this investigation are *Candida tropicalis* (MTCC 230), *Candida albicans* (MTCC 183), *Candida glabrata* (MTCC 6507) and *Candida parapsilosis* (MTCC 6510). These *Candida* species were sub-cultured on sabouraud dextrose agar (SDA) at 30°CC for 16 h and maintained at 4°CC in a refrigerator for short-term storage. For long-term storage, the strains were stored as frozen stock with 15% (v/v) glycerol at –80°C. For experiment purposes, *Candida* cells were cultured in sabouraud dextrose broth (SDB) in a shaking incubator at 30°C.

### Inoculum preparation

Throughout the experiment, we used fresh cultures by sub-culturing each *Candida* species separately on SDA plates at 30°C for 24-48 h. The *Candida* species were first grown on SDA and then transferred to test tubes containing 0.9% sterile saline (5 ml). The strains were then individually diluted in sterile saline (0.9%) until they reached a 1×10^5^ CFC/ml concentration, which was then compared to the McFarland scale. This technique provided a standard *Candida* suspension (1×10^5^ cells/ml), which is used for the following assays.

### Determination of minimum inhibitory concentration of the seed extracts

The MIC of *C. bonduc* seed extracts was resolved using the micro-broth dilution technique depicted by the Clinical and Laboratory Standards Institute (CLSI, USA) with a little modification (CLSI, 2008). To determine the MIC, the 96-well sterile ELISA plates were first filled with SDB (100 μl), followed by a serial dilution of three *C. bonduc* seed extracts and amphotericin-B (positive control). The standard *Candida* ****inoculum (100 μl) was then added to the plates, resulting in a final concentration range of 16–6000 µg/ml for *C. bonduc* seed extracts and 0.12 to 500 µg/ml for amphotericin-B. Microtiter plates were covered with paraffin film (Tarson’s products) and incubated at 30°C for 24 h. MIC values were noted based on the visual observation of the plates after the incubation period. In addition to visual observation, we also verified the MIC by measuring the OD spectrophotometrically. The MIC was defined as the lowest concentration of the test material (plant extract) at which no growth was observed. The experiment was performed in triplicate sets.

### Determination of minimum fungicidal concentration of the seed extracts

The MFC of**** *C. bonduc* seed extracts was assessed as previously described (Shokri and Sharifzadeh, 2017). At the end of 24 h of incubation at 30˚C, an aliquot of 10 µl from each well having no visible growth in the MIC experiment was serially diluted in sterile saline (0.9%) and plated on SDA plates and incubated for 48 h at 30˚C. After the incubation period, the colonies of *Candida* grown on SDA** **plates were counted. MFC is defined as the lowest concentration of test substance that results in complete fungal death (99.9%) when compared to the initial inoculum. These assays were also executed in triplicate sets.

### Characterization of bioactive compounds in EECS through LC-QTOF-MS analysis

The characterization of bioactive compounds present in EECS was carried out by LC-QTOF-MS (Agilent Technologies, Santa Clara, CA, USA, Model no: 6545 QTOF).

### Determination of time kill curve of EECS

*C. albicans* suspension was revamped to a 0.5 McFarland turbidity standard with a cell density of approximately 1×10^6^ CFU/ml. To determine the time-kill kinetics of EECS, *C. albicans* was treated over time with three different concentrations of EECS (0.5× MIC, MIC and 2× MIC) and then incubated at 30°C in a shaking incubator. The required samples were withdrawn aseptically after being vortexed at fixed time intervals (0, 2, 4, 6, 12, 24, and 48 h) to determine the colony count. After that, the samples were subjected to serial dilution in sterile saline and then plated onto SDA plates using a sterile L-rod. Subsequently, plates were incubated at 30°C for 48 h. Here, amphoteric-B serves as the standard drug control, whereas the medium without EECS serves as the normal control. All the tests were performed in triplicate.

### Inhibition of biofilm formation by EECS

*C. albicans* biofilms were grown on the 96-well flat-bottomed ELISA plates and the potential of EECS to inhibit the formation of biofilm was measured by calculating the reduction of 2,3-bis(2-methoxy-4-nitro-5-sulfo-phenyl)-2H-tetrazolium-5 carboxanilide (XTT) by adopting the protocol reported earlier (Kim et al., 2017). First, *C. albicans* suspensions were prepared at 1×10^6^ cells/ml in SDB. An aliquot (100 μl) of suspension was transferred into microtiter plates and treated with three different concentrations of EECS (0.5× MIC, MIC and 2× MIC) in a final volume of 200 μl/well. Then the plates were incubated at 30°C for 24 h. The biofilms were washed twice with PBS after 48 h at 30°C, and the inhibition of biofilm formation by the cells was measured using a standard XTT reduction assay. The XTT reduction assay measures mitochondrial dehydrogenase activity, which will be displayed by intact biofilms only (Chandra et al., 2008). Additionally, the inhibition of biofilms after the EECS treatments was inspected using a microscope (BD Pathway™ Bioimager system, USA).

### Determining the effect of EECS on reactive oxygen species production in *C. albicans* cells

2, 7’- dichlorofluorescein diacetate (DCFH-DA) staining method was adopted to verify ROS production in *C. albicans* cells after treating with EECS (Jia et al., 2015). Briefly, log-phased *C. albicans* cells (1×10^6^ cells/ml) were cultured in SDB at 30°C for 24 h and then centrifuged for 10 min at 5000 rpm. The *C. albicans* pellet thus obtained was again resuspended in PBS comprising 0.5× MIC, MIC and 2× MIC concentrations of EECS in test tubes. H_2_O_2_ (2.5 mM) serves as the positive control and amphotericin-B (1 µg/ml) as the standard drug control. The tubes were incubated at 30°C for 60 min. After incubation, 10 μM 2,7-dichlorofluorescin diacetate (DCFH-DA) in PBS was added. The fluorescence intensities of *C. albicans* cells were investigated using FACSVerse flow cytometry (Becton Dickinson, San Jose, CA, USA).

### Evaluation of the cell membrane integrity through a propidium iodide uptake assay after EECS treatment

The effect of EECS on cell membrane integrity was investigated by means of a PI uptake analysis. For this, *C. albicans* (1×10^6^ CFU/ml) were exposed to 0.5× MIC, MIC and 2× MIC concentrations of EECS and incubated for 3 h at 30°C in a shaking incubator (120 rpm). H_2_O_2_ (2.5 mM) serves as the positive control and amphotericin-B (1 µg/ml) as the standard drug control. After incubation, the *C. albicans* suspension was mixed with the PI solution (50 mg/ml) and incubated for 10 minutes at 30°C with the tubes covered with aluminium foil. Afterwards, the *C. albicans* cells were cleaned with PBS buffer. Then the samples were subjected to microscopic examination using a fluorescence microscope (BD Pathway™ Bioimager system, USA), with suitable fluorescent filters (excitation wavelength: 530 nm and emission wavelength: 590 nm) (Seyedjavadi et al., 2020). Here, the cells without treatment serve as the negative control. The experiments were conducted in triplicate sets.

### Analysis of apoptosis by Annexin V staining

For Annexin V staining, cells were first thoroughly washed using 0.1 M PPB (Potassium Phosphate Buffer) and again suspended in PPB buffer comprising sorbitol (1 M) and lysing enzyme (20 mg/ml). Thus, the obtained suspensions were incubated for 4 h at 30°CC with mild shaking (60 rpm) in a shaking incubator. Subsequently, protoplast cells were obtained by filtration followed by centrifugation for 10 min at 1500 rpm. The protoplasts obtained were then exposed to 0.5× MIC, MIC and 2× MIC concentrations of EECS and incubated at 30°CC for 3 h. Here, H_2_O_2_ (2.5 mM) serves as the positive control and amphotericin-B (1 µg/ml) as the standard drug control. Following incubation, the cells were cleaned with Annexin V binding buffer comprised of sorbitol (1 M). After that, the cells were subjected to incubation with 5 µg/ml of Annexin V-FITC and PI using an Annexin V-FITC Apoptosis Detection Kit (Sigma-Aldrich, USA) at normal room temperature for 15 min. The stained *Candida* cells were observed using fluorescence microscopy (BD Pathway™ Bioimager system, USA). The experiment was performed in triplicate.

### Measurement of mitochondrial membrane potential (mtΔψ)

The mitochondrial membrane potential (MMP, mtΔψ) is a vital pointer to knowing the structure and function of mitochondria; thus, we investigated the outcome of EECS treatment in *C. albicans* by verifying mtΔψ variation using an earlier reported procedure with minor alterations** ** (Li et al., 2020). For this, *C. albicans* (1 × 10^6^ cells/ml) were exposed to 0.5× MIC, MIC and 2× MIC concentrations of EECS** **in SDB medium for 4 h at 30˚C. Here also, H_2_O_2_ (2.5 mM) serves as the positive control and amphotericin-B (1 µg/ml) as the standard drug control. After incubation, the cells were stained with 5 μM Rhodamine-123 (Rh-123) for 30 min in the dark. The fluorescence intensity of the treated cells was examined after three washes with sterile PBS using a spectrofluorophotometer (Shimadzu UV-1700) (excitation: 486 nm and emission: 525 nm), and further Rh-123 stained cells were examined with the help of a fluorescence microscope (BD Pathway™ Bioimager system, USA).

### Detection of nuclear and DNA disruption due to the treatment with EECS

TUNEL and 6-diamidino-2-phenylindole (DAPI) staining methods were employed to examine DNA fragmentation and condensation after EECS treatment (Hwang et al., 2014). *C. albicans* (Log-phase) was cultivated in SDB and then resuspended in PBS. After that, the *Candida* cells were subjected to 0.5× MIC, MIC and 2× MIC concentrations of EECS. H_2_O_2_ (2.5 mM) serves as the positive control and amphotericin-B (1 µg/ml) as the standard drug control. Afterwards, *Candida* cells were cleaned thrice with PBS buffer and then fixed in paraformaldehyde (3.6%) for 25 min, and further permeabilized in a permeabilization solution [Triton X-100 (0.1%) and sodium citrate (0.1%)] for 2 min on ice. After this, the *Candida* cells were washed again with PBS and subjected to the TUNEL assay by using the detection kit (Sigma-Aldrich, USA) for 1 h at 30°CC by following the instructions provided by the manufacturer to determine DNA fragmentation. The stained cells were examined with the help of a fluorescent microscope (BD Pathway™ Bioimager system, USA) (excitation: 358 nm and emission: 461 nm).

Nuclear condensation was studied by using DAPI staining (Sigma–Aldrich, USA). Mid-log phased *C. albicans* (1 × 10^6^ CFU/ml) were exposed to 0.5× MIC, MIC and 2× MIC concentrations of EECS for 6 h at 30°C. *C. albicans* that weren’t treated with EECS served as a negative control. H_2_O_2_ (2.5 mM) serves as the positive control and amphotericin-B (1 µg/ml) as the standard drug control. After treatment, *C. albicans* cells were cleaned with PBS (10 mM, pH 7.2) and then stained with DAPI (50 µg/ml) for 20 minutes in the dark. Subsequently, the cells were examined with the help of a fluorescent microscope (BD Pathway™ Bioimager system, USA) at 358 nm of excitation and 461 nm of emission.

### Outcome of EECS treatment on the discharge of *cytochrome c*

The outcome of EECS on *cytochrome c* release in *C. albicans* cells was investigated by following a previously reported protocol (Wani et al., 2021). Briefly, *Candida* cells (1 × 10^6^ CFU/ml) were treated with 0.5× MIC, MIC and 2× MIC concentrations of EECS and then incubated in a shaking incubator for 4 h at 30˚C. H_2_O_2_ (2.5 mM) serves as the positive control and amphotericin-B (1 µg/ml) as the standard drug control. After treatment, the *Candida* cells were centrifuged, cleansed with fresh PBS, and then further subjected to homogenization in buffer A solution [EDTA (0.002 M), Phenylmethylsulfonyl fluoride (0.001 M), Tris base (0.05 M, pH 7.5)]. Subsequently, it was subjected to a next round of centrifugation for 10 min at 4000 rpm. The supernatant thus obtained was carefully collected in clean test tubes separately and again subjected to centrifugation at 50,000 rpm for 40 min. After centrifugation, the supernatant was collected in a clean vial and subjected to estimation of the level of *cytochrome c* in the cytosolic. The leftover pellet was carefully dissolved in buffer B [EDTA (0.002 M), Tris base (0.05 M; pH 5.0)] and used for assessing the mitochondrial *cytochrome c* level. The *cytochrome c* present in the cytosol and mitochondria was lowered by adding ascorbic acid (500 mg/ml) and further analyzed by a spectrofluorophotometer (Shimadzu UV-1700) at 550 nm.

### Determination the calcium level in mitochondrial and cytosolic after EECS treatment

The treatment effects of EECS on the Ca^2+^ level in the cytosol were investigated using Fluo-3 AM, whereas the mitochondrial Ca^2+^ level was estimated with the aid of Rhod-2 AM (Tian et al., 2017a). *C. albicans* was exposed to different concentrations of EECS and the cells were collected by centrifugation to determine the Ca^2+^ level. Here, H_2_O_2_ (2.5 mM) serves as the positive control and amphotericin-B (1 µg/ml) as the standard drug control. After that, the cells were cleaned twice with Hanks’ Balanced Salt Solution (HBSS) and again suspended in HBSS (500 µl). In order to calculate the cytosolic Ca^2+^ level, Fluo- 3 AM was added to the resuspended cells at a final concentration of 2 µM. Subsequently, the cells were incubated at 30°CC for 40 min in dark conditions. After incubation, the cells were cleaned again and then resuspended in HBSS (600 µl). Afterward, the cells were subjected to incubation again for 20 min at 30°CC. In order to quantify the cytosolic Ca^2+^ level, *Candida* cells were stained with Fluo-3 AM (2 mM) for 40 min at 30°CC in dark conditions. After this, the *C. albicans* cells were rewashed and suspended again in HBSS (600 μl) and subjected to incubation for an additional 15 min at 30°CC. The fluorescence intensity was quantified using a spectrofluorophotometer (Shimadzu UV-1700). For Fura-2AM, the excitation wavelength employed is 340 nm, whereas the emission wavelength is 510 nm. The fluorescence intensity in the case of Rhod-2AM was measured at an excitation wavelength of 550 nm and an emission wavelength of 580 nm.

### Caspase activation assay

The activation of caspase after EECS treatment was assessed using CaspACE FITC-VAD-FMK (FITC conjugate of the cell-permeable caspase inhibitor VAD-FMK). Moreover, this structure permits the inhibitor to enter the cell and then bind to activated caspase, which acts as a strong apoptotic indicator. The *C. albicans* (1×10^6^ CFU/ml) were exposed to 0.5× MIC, MIC and 2× MIC concentrations of EECS for 4 h at 30°CC. H_2_O_2_ (2.5 mM) serves as the positive control and amphotericin-B (1 µg/ml) as the standard drug control. After that, the cells were subsequently cleaned with PBS and then stained with CaspACE FITC-VAD-FMK (2.5 μM). Afterwards, stained *Candida* cells were examined using a fluorescence microscopy (Li et al., 2020). The cells were further analyzed with the help of a spectrofluorophotometer at an excitation wavelength of 490 nm and an emission wavelength of 525 nm for FITC-VAD-FMK (Lee and Lee, 2018).

### In-vivo antifungal potential of EECS in *Galleria mellonella*–*C. albicans* infection model

*G. mellonella* larvae were continuously* *maintained in the laboratory and were fed on an artificially prepared diet (Wheat flour 350 g, corn flour 200 g, milk powder 130 g, backing yeast powder 70 g, honey 100 ml, and glycerin 150 ml)* * (Metwally et al., 2012). Final instar *G. mellonella* larvae (weights: 270 to 300 mg) were selected for conducting this assay. To study the in-vivo effect of EECS, survival assays of *G. mellonella* after infection with *C. albicans* were conducted based on a methodology reported earlier (Li et al., 2019). For this assay, 5×10^8^ CFU/ml concentration of* C. albicans* was obtained by diluting in sterile PBS. EECS at a concentration of 0.5× MIC, MIC and 2× MIC was used for the survival assay. The standard drug used for this experiment was amphotericin-B (1 μg/ml). *G. mellonella* larvae weighing 220–280 g were chosen and assigned into six groups (control, 0.5× MIC, MIC, 2× MIC and amphotericin-B). Each group was comprised of 20 larvae. After that, *C. albicans* (10 µl) was administered into the proleg of larvae (last left proleg) *via* injection. Subsequently, the injected larvae were incubated at 37°C for 2.5 h in sterile petri dishes. After successful *Candida* infection, 10 µl sterile PBS was injected into the control group, whereas the other groups received 10 µl of the EECS and amphotericin-B in the last right proleg. Subsequently, the larvae were kept at 37°C in the dark room to observe the mortality at various time periods. The assay was conducted in three replications.

### Cytotoxicity assessment of EECS

The cytotoxic potential of EECS was assessed using horse red blood cells by adopting a previously explained method (Lone et al., 2020), with slight variations. Briefly, horse blood (10 ml) was aliquoted into sterile falcon tubes (50 ml) and rotated for 10 min at 2000 rpm. Subsequently, the obtained cell pellet was cleaned thrice using a chilled PBS solution and a 10% RBC suspension was obtained by suspending the cells again in a cold PBS solution. This RBC suspension was again diluted 10 times using a PBS solution. Thus, the obtained RBC suspension (100 µl) was exposed to different concentrations of EECS (0.5× MIC, MIC and 2× MIC) along with a positive control. The treated RBC suspension was subsequently kept for 1 h at room temperature, which was followed by rotation at 2000 rpm for 10 min. The 200 µl of the supernatant thus obtained was added to the wells of an ELISA plate (Falcon, USA) and the absorbance was recorded with the help of an ELISA plate reader at 450 nm (Molecular Devices, USA). In this study, the positive control used was Triton X-100 (1%), whereas the negative control was PBS. The outcome was expressed in terms of the percentage of hemolysis and was determined using the following formula:

$$\%\;of\;haemolysis=\frac{\left[\left(A450\;of\;treated\;sample\right)-\left(A450\;of\;negative\;control\right)\right]}{\left[\left(A450\;of\;positive\;control\right)-\left(A450\;of\;negative\;control\right)\right]}\times 100$$

### Statistical analysis

All the assays were conducted in triplicate set and are expressed as the mean ± standard deviation (SD). The change of data between the treated groups and controls was carried out by applying the Simple Analysis of Variance (ANOVA) followed by *Tukey* test (95% confidence interval), using SPSS and *p* < 0.05 was considered significant.

## Results

### Anti-candida activity of *C. bonduc* seed extracts

This study used various concentrations of *C. bonduc* seed extracts to assess the MIC and MFC against four *Candida* species. In our study, the MIC values of the extracts were recorded from 64 to 4000 µg/ml, while MFC values were recorded from 125 to 4000 µg/ml (**Table 1**). In case of amphotericin-B, MIC and MFC values were recorded at 0.5 to 1 µg/ml (**Table 1**). The best activity was recorded by ethanolic extract of *C. bonduc* seed (EECS) against *C. albicans *(64 µg/ml), followed by *C. tropicalis* (125 µg/ml)*.*The aqueous extract didn’t exhibit any activity against tested *Candida* species.

**Table 1:** MIC and MFC of *C. bonduc* seed extracts against *Candida* species.

Test fungi	MIC/MFC (µg/ml)			
	Ethanol	Hydroalcoholic	Aqueous	Amphotericin-B
*C. albicans*	64/125	250/500	–	0.5/1
*C. tropicalis*	125/250	1000/2000	–	1/1
*C. glabrata*	1000/1000	2000/4000	–	0.5/1
*C. parapsilosis*	1000/2000	4000/4000	–	1/1

- No activity up to 6000 µg/ml.

From the MIC and MFC results, it was clear that out of three extracts tested, only EECS recorded significant anticandidal activity against *C. albicans.* Thus, we selected EECS for a detailed investigation regarding the mechanism of action against *C. albicans.*

### Identification of compounds present in EECS

**Figure 1:**
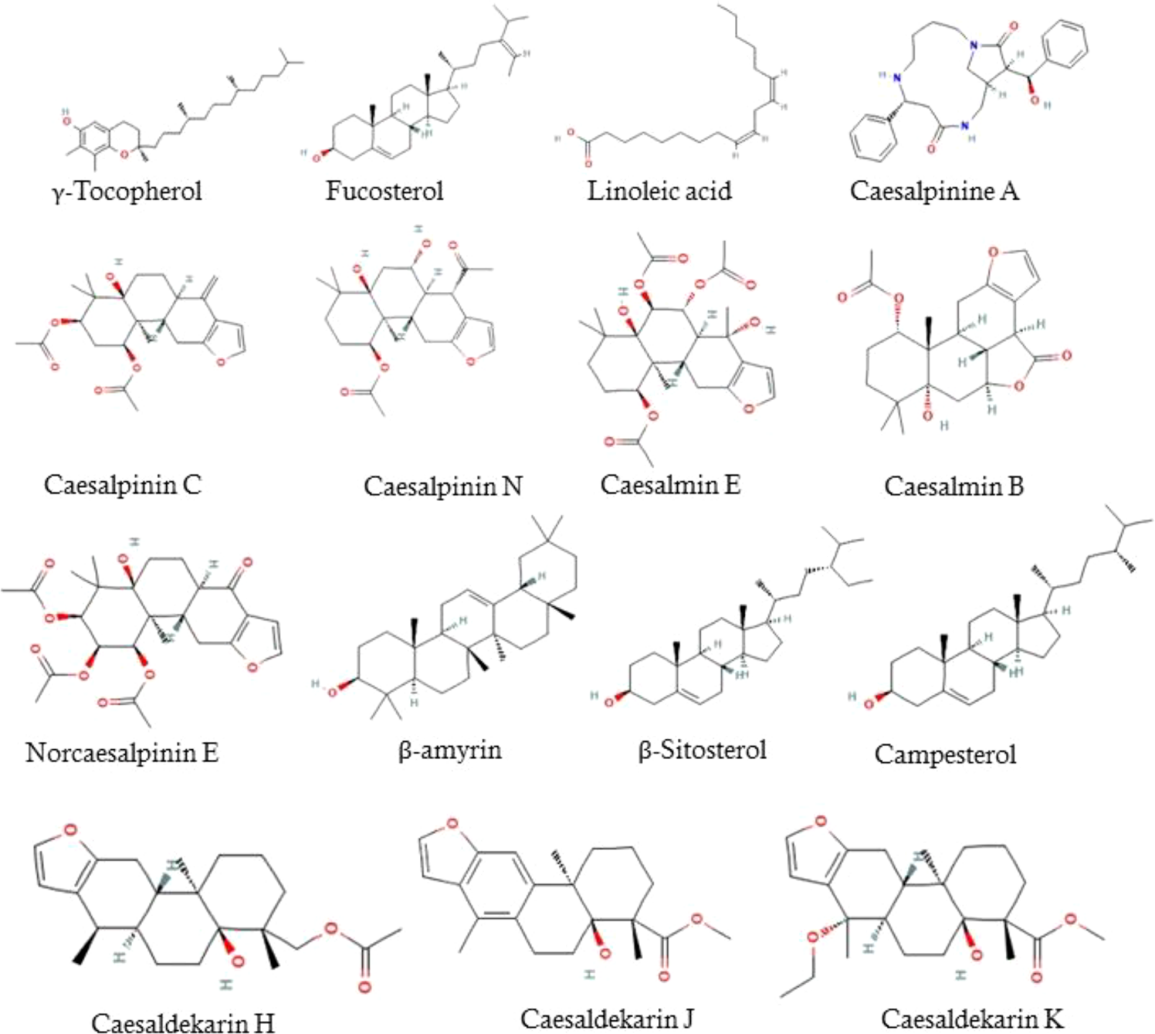
Bioactive compounds present in EECS.

The LC/MS-QTOF analysis recorded that the major compounds present in the ethanolic extract was Tocopherols, Linoleic acid, Caesalpinines, Caesalmins, Norcaesalpinins, β-amyrin, β-Sitosterol, Campesterol, Caesaldekarins (**Figure 1**).

### EECS recorded significant rates of killing in time-kill experiment

**Figure 2:**
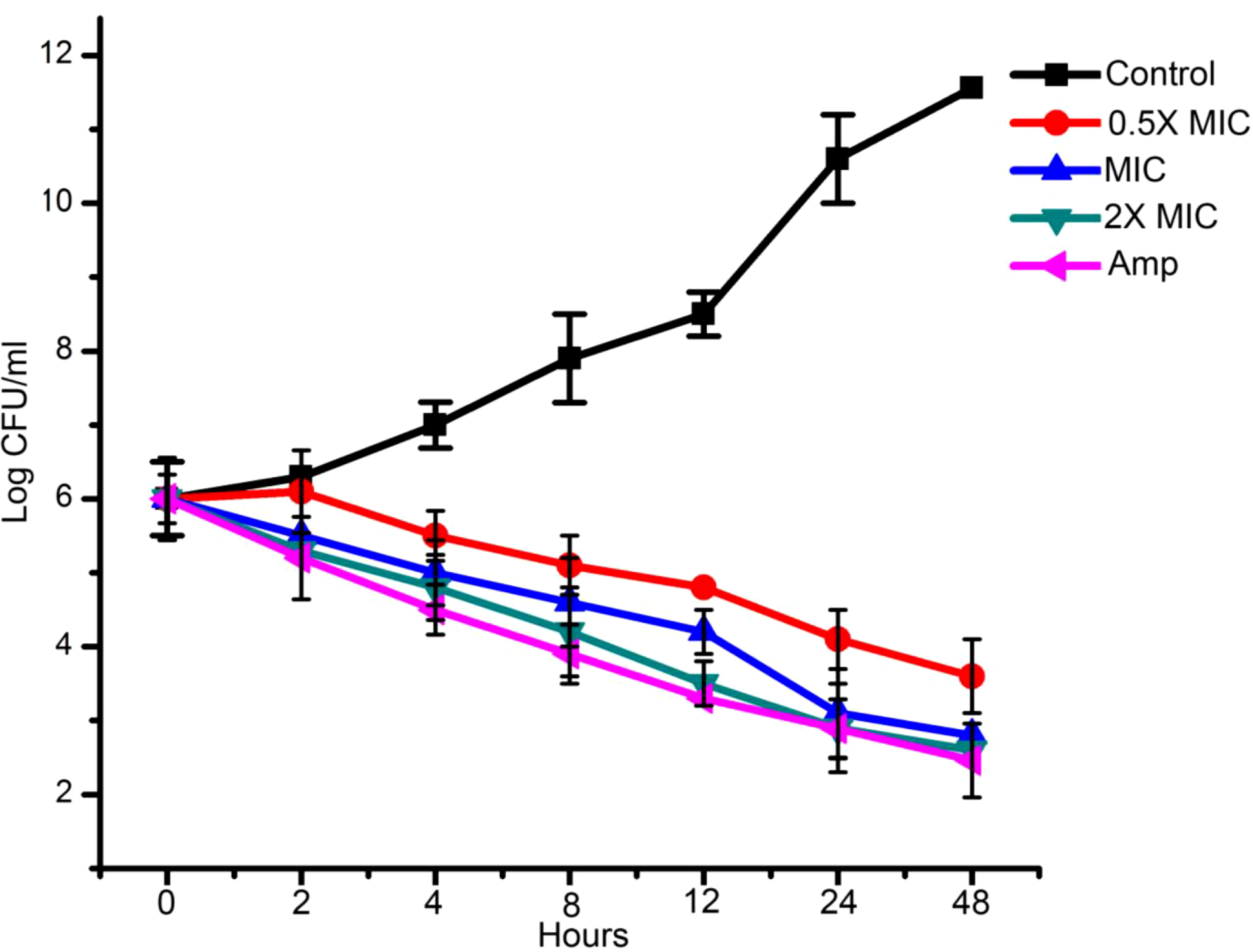
Time kill assay of EECS against *C. albicans.* Data are expressed as the mean ± standard deviation. Data represent the results of three independent experiments. (p < 0.05).

The effect of EECS against *C. albicans* was further established by conducting a time-kill curve assay. **Figure 2** shows the time-kill result of EECS on *C. albicans*. This experiment was performed to confirm the killing rates of *C. albicans* when treated with EECS (**Figure 2**). The maximum reduction of *C. albicans* growth by the EECS was recorded between 4 and 12 h. At 48 h, the EECS recorded a 99.99% reduction of the initial inoculum of *C. albicans*. Here in this study, 2× MIC concentration of EECS recorded a significant effect, which is comparable with that of the standard drug amphotericin-B. Regrowth was not observed for *C. albicans* treated with EECS.

### EECS significantly inhibits the biofilm development of *C. albicans* in in-vitro conditions

**Figure 3:**
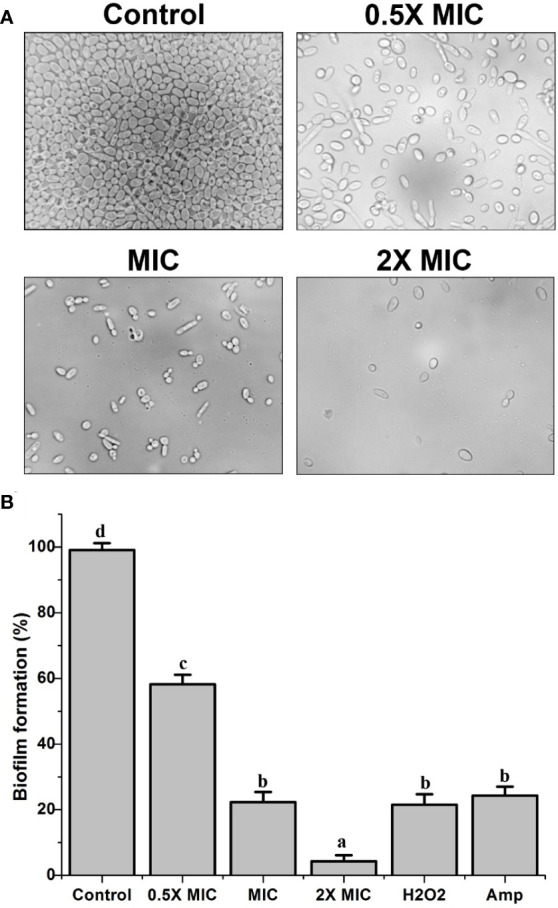
The inhibitory activity of EECS against *C albicans* biofilms. **(A)** Inhibition of biofilm by EECS when observed through microscopy. **(B)** The result of XTT reduction assay regarding the inhibition of EECS on biofilm formation. The experiment was performed in triplicate sets. The average followed by different letters are statistically different (p < 0.05).

The biofilm formation by *C. albicans* is renowned as a vital factor in yeast infections in humans, especially in implanted medical devices. Besides this, biofilm formation is also related to severe drug resistance and associated pathogenicity. XTT reduction assays were conducted to examine whether EECS affects *C. albicans* biofilm formation. The results from this experiment clearly confirmed a remarkable dose-dependent reduction in the biofilm formation by *C. albicans* (**Figure 3A**). At its MIC concentration, EECS lowered the metabolic potential required for the biofilm formation of *C. albicans *by more than 75% (**Figure 3B**). On the other hand, a 2× MIC concentration of EECS reduced biofilm formation by more than 95%. The amphotericin-B (1 μg/ml) also recorded a noticeable reduction in the formation of biofilm. The microscopy examination revealed that EECS treatment ended in a noteworthy defect in the formation of biofilm when compared with the control sample (**Figure 3A**). In addition to this, we observed that EECS effectively inhibits the yeast-to-hyphae transition while inspecting the cell morphology through the microscope. The potential of EECS to inhibit biofilm formation was also an apparent effect of its significant fungicidal activity.

### Treatment with EECS significantly increases the intracellular ROS generation in *C. albicans*

**Figure 4:**
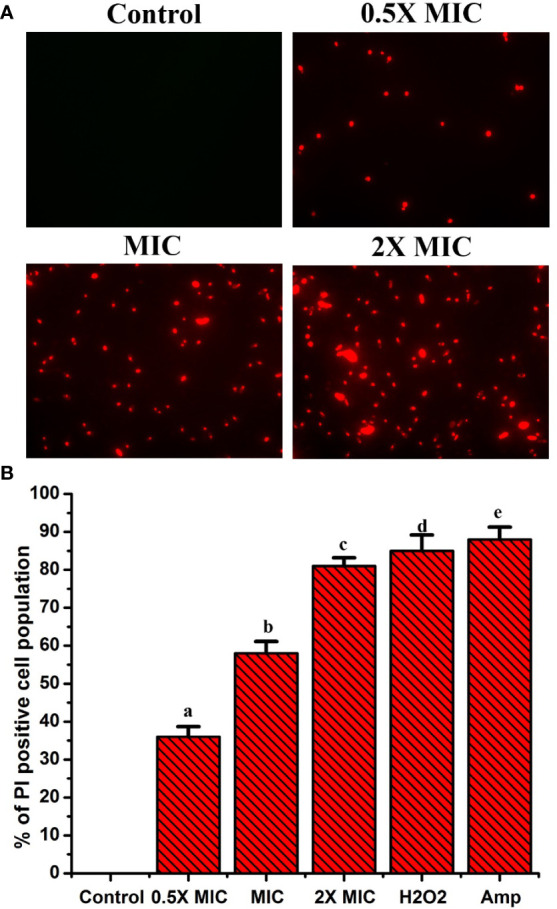
The treatment of EECS significantly enhanced the production of ROS in *C albicans*. **(A)** Treatment with EECS induced ROS production in *C albicans* cells when observed using a flow cytometer. **(B)** The percentage of DCFH-DA stained cells indicated the presence of ROS when treated with different concentrations of EECS. The positive control used was H_2_O_2_ (2 mM). The experiment was performed in triplicate sets. The graph represents average ± standard deviation values. The average followed by different letters are statistically different (p < 0.05).

ROS formation is one of the effective antifungal mechanisms of several plant extracts with antifungal activity. The generation of ROS by EECS was examined after incubating EECS at various concentrations with the *Candida* cells. The fluorescence, which results from the oxidation of DCFH-DA (non-fluorescent molecule) dye into DCF (fluorescent molecule), was recorded through flow cytometry, demonstrating the presence/generation of ROS after the drug treatment. The percentage of DCF stained cells confirmed the ROS production by the EECS (**Figure 4**). As depicted in **Figure 4**, the cells exposed to EECS recorded a considerable upsurge in the level of intracellular ROS when related to the untreated control sample, suggesting that EECS leads to ROS accumulation in *C. albicans*. In addition, results from our study clearly demonstrated that the EECS enhanced the production of ROS in a dose-dependent manner (**Figure 4**).

### EECS severely affects the cell membrane permeability of *C. albicans*

**Figure 5:**
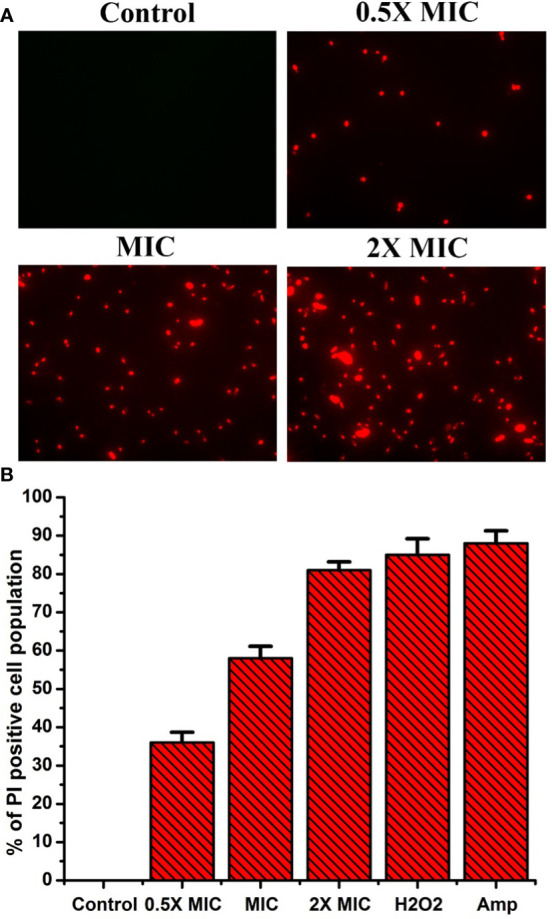
PI uptake assay shows that the treatment with EECS in *C albicans* significantly affects the permeability of cell membrane. **(A)** Fluorescence microscopy investigation of cell membrane damage was clearly evident by the red colored *Candida* cells in PI uptake assay after EECS treatment. **(B)** The percentage of PI-positive *Candida* cells after treatment with various concentrations of EECS. To get the percentage of PI-positive *Candida* cells, the PI-positive cells were counted against the normal cells in 10 different fields. H_2_O_2_ (2 mM) was employed as a positive control and amphotericin-B (Amp) was employed as a drug control. The experiment was performed in triplicate sets. The graph represents average values ± standard deviation. The average followed by different letters are statistically different (p < 0.05).

Propidium iodide (PI) is a fluorescent dye that intercalates DNA but does not enter intact healthy cells. The PI enters the cytoplasm once the cell membrane is injured, leading to DNA binding. Thus, PI can be used to explore the cell membrane integrity of *C. albicans* after treatment with any drug. PI dye can pass only through damaged membranes for binding with DNA, which produces a bright red fluorescence when examined under a fluorescence microscope. So, we used a PI uptake assay to investigate the cell membrane integrity of *C. albicans* after EECS treatment. The PI stained *C. albicans* after treatment with various concentrations of EECS is shown in **Figure 5**. The fluorescence microscope analysis recorded that the number of deceased *C. albicans* increases when the concentration of EECS increases from 0.5× MIC to 2× MIC concentration. In this study, the PI uptake assay showed a noteworthy increase in the PI-positive cell population after EECS treatment when compared with the control cells, suggesting that the EECS triggered cell membrane damage of *C. albicans*.

### EECS significantly increased the exposition of phosphatidylserine (PS)- a hallmark feature of apoptosis

**Figure 6:**
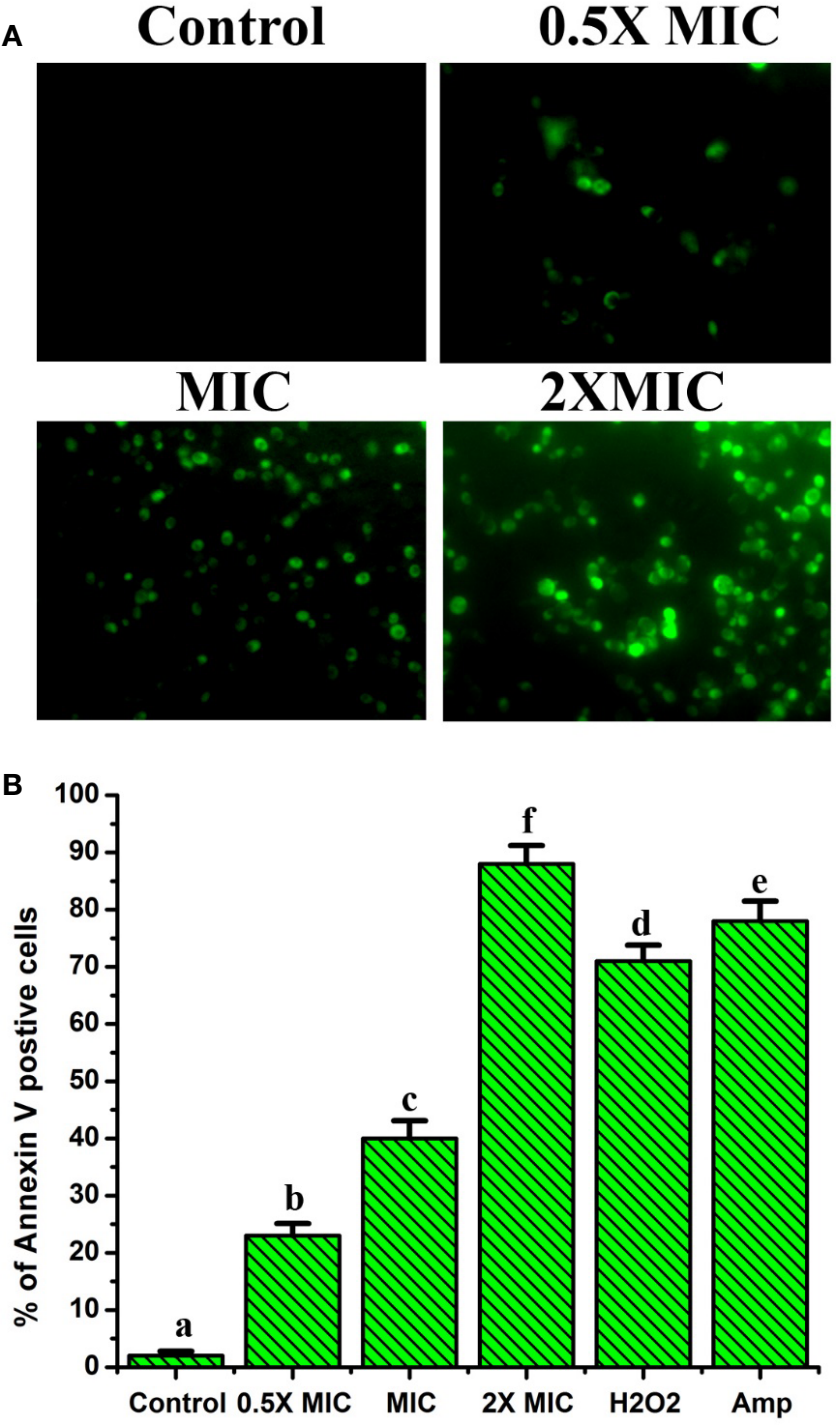
Annexin V and PI staining of *C albicans* after EECS treatment. **(A)** Fluorescence microscopy investigation of PS externalization as evident by the green colored *Candida* cell. **(B)** Percentage of Annexin V positive *Candida* cell after exposed with various concentrations of EECS. Positive control used was H_2_O_2_ (2 mM) and amphotericin-B (Amp) was used as a drug control. The experiment was performed in triplicate sets. The graph represents average ± standard deviation values. The average followed by different letters are statistically different (p < 0.05).

In yeasts (Candida), PS is mainly found on the inside leaflet of the lipid bilayer of the cytoplasmic membrane, as it is in mammalian cells, and is translocated to the outside of the leaflet at the time of early apoptotic stages (Wu et al., 2010). For this reason, exposure to PS serves as a vital hallmark for identifying the early apoptotic phase. This can be determined with the aid of Annexin V staining. In this method, green-stained cells indicate early apoptotic and late-apoptotic/necrotic cells will be marked green and red, whereas necrotic cells will be stained only with red color. Thus, we used FITC-labeled annexin V and PI staining method to study the level of PS externalization in *C. albicans* after EECS treatment. **Figure 6** shows annexin V and PI staining results after treating EECS with different concentrations. *C. albicans* treated with EECS predominantly stained green, demonstrating the occurrence of PS at the outside surface of the cytoplasmic membrane, which is clear evidence of early apoptotic events induced by the EECS (**Figure 6**). No necrotic cells were observed in the study. This result suggests that** **EECS exposed *C. albicans* cells mostly endure apoptosis rather than necrosis.

### EECS significantly enhanced the mtΔψ in *C. albicans*, demonstrating the dysfunction and destruction of mitochondria

**Figure 7:**
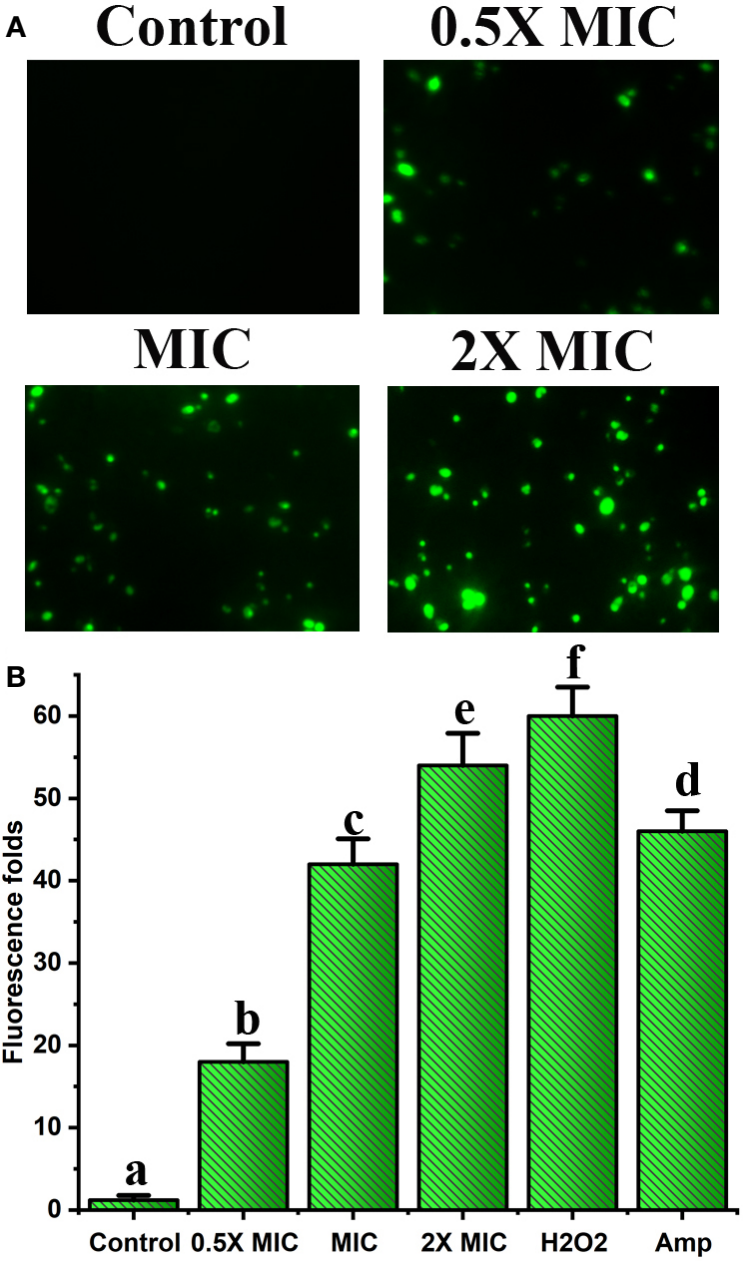
Effect of EECS on mitochondrial membrane potential. **(A)** Fluorescent microscopic examination of Rh-123 stained *C albicans* exposed to various EECS concentrations. **(B)** Spectrofluorophotometric analysis of the increase in relative fluorescence intensity of *C albicans* cells after EECS treatment. H_2_O_2_ (2 mM) was employed as a positive control and amphotericin-B (Amp) was used as a drug control. The experiment was performed in triplicate sets. The average followed by different letters are statistically different (p < 0.05).

The mtΔψ is a strong pointer of mitochondrial energetic state. Moreover, mtΔψ can be employed to evaluate the bustle of proton pumps in mitochondria, electron transport systems, and the triggering of the permeability of mitochondria due to various origins (Li et al., 2015). Furthermore, in aerobic** **cells, the mitochondrial apparatus has long been recognized as a key source for the generation of ROS. Hence, we studied the outcome of EECS on the function of mitochondria by studying the** **alteration of mtΔψ. Rh-123 was employed to examine the mtΔψ of EECS exposed C. albicans. The results of fluorescence microscopic analysis displayed a dose-dependent increase in the number of *C. albicans *with green stain (**Figure 7**). The intensity of fluorescence measured for the EECS-treated cells was also positively related to the increased doses of EECS. Juxtaposed with the EECS-free control, the extract treated samples significantly increased fluorescence levels by 18.1, 42.56 and 54.22-fold, respectively (**Figure 7**). These results suggest that EECS treatment increased the mtΔψ in *C. albicans*, which indicates the mitochondrial dysfunction due to the treatment with EECS.

### Treatment with the EECS produces morphological changes in both DNA and nuclei

**Figure 8:**
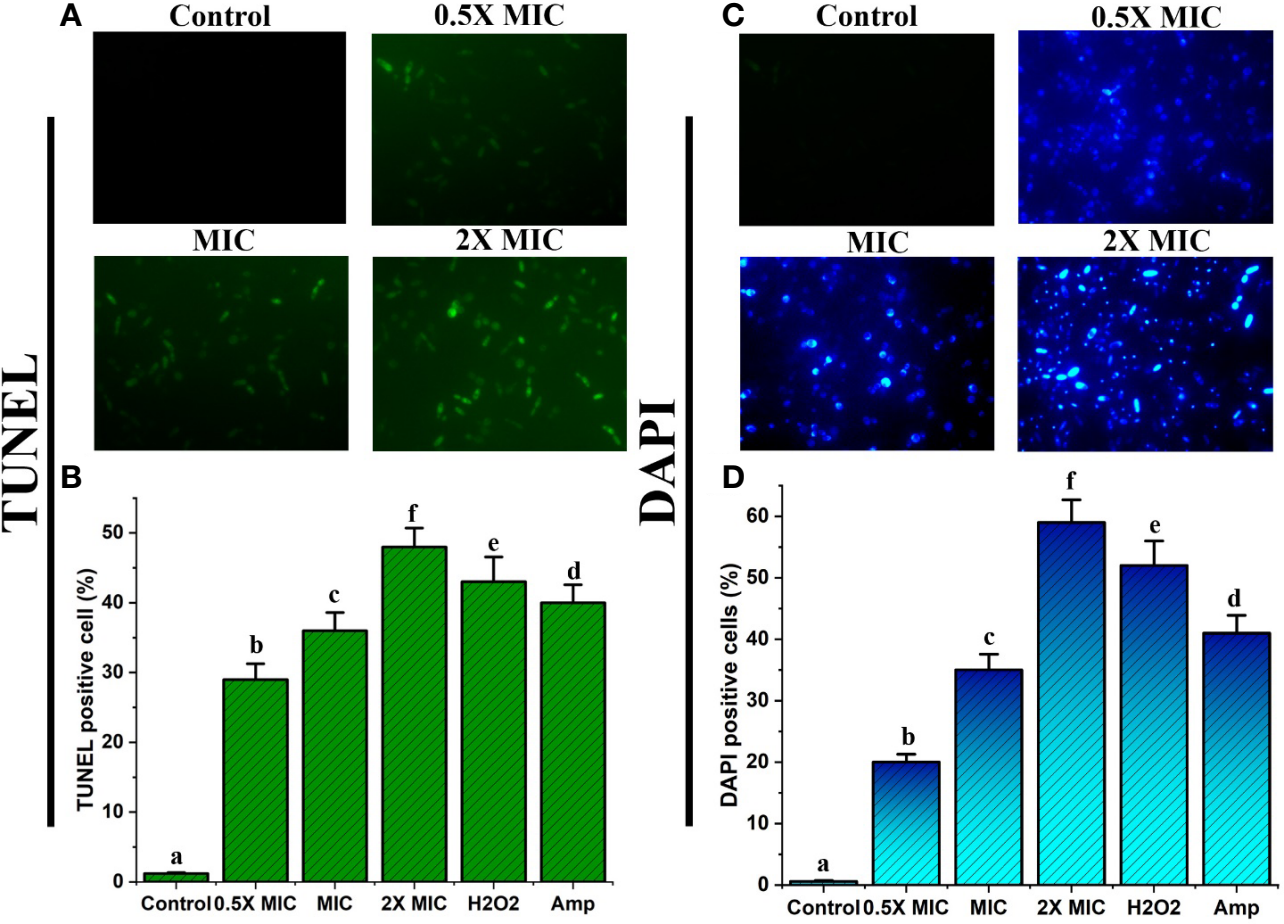
EECS exerted significant nuclear and DNA damage in *C albicans* cells. **(A)** TUNEL staining of *C albicans* after EECS treatment and fragmentation of DNA was envisaged as green fluorescence. **(B)** The percentage of TUNEL-positive *C albicans*. **(C)** Nuclear fragmentation observed in *C albicans* cells treated with EECS while detected using DAPI staining. **(D)** The percentage of DAPI-positive *C albicans* cells. H_2_O_2_ (2 mM) was used as a positive control and amphotericin-B (Amp) was used as a drug control. The experiment was performed in triplicate sets. The average followed by different letters are statistically different (p < 0.05).

Nuclear DNA degradation generates free 3’-OH. Exposed free 3’-OH produced by fragmented DNA reacts with FITC-labeled dUTP under the catalysis of terminal deoxynucleotidyl transferase (Li et al., 2019). ROS may be an important factor for the fragmentation of DNA and is observed mainly in apoptotic cells. In this study, the TUNEL assay displayed intensive green fluorescence in* C. albicans *treated with various concentrations of EECS when compared with the control cells (**Figure 8A**). These results clearly show that the DNA strand has been broken due to fragmentation. In addition to this, the EECS treated *Candida* cells recorded a substantial increase in the number of TUNEL-positive cells compared to the untreated control cells (**Figure 8B**).

Here, we used a DAPI staining assay to confirm the morphological variations in the nucleus of *Candida* cells after EECS treatment. DAPI is used to confirm the morphological changes where it mainly binds the minor groove of the A: T rich region of the DNA sequences. Fluorescence microscopy examinations disclosed that EECS treated *C. albicans* recorded significant fluorescence when compared to the control *C. albicans* cells (**Figures 8C, D**). This result established that EECS significantly damaged the nucleus, which led to chromatin condensation and further nuclear fragmentation.

Overall, the results of the TUNEL and DAPI assays specified that EECS treatment in *C. albicans* led to nuclear condensation and DNA fragmentation, hallmark properties exhibited during apoptotic cell death.

### Treatment with EECS significantly induced mitochondrial *cytochrome c* discharge in *C. albicans*

**Figure 9:**
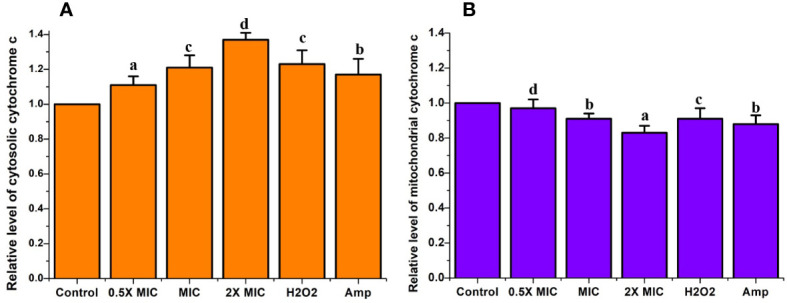
The *cytochrome c* release in *C albicans* after EECS treatment. **(A)** The level of relative fluorescent value of cytosolic *cytochrome c*. **(B)** The level of relative fluorescent value of mitochondrial *cytochrome c* H_2_O_2_ (2 mM) was employed as a positive control and amphotericin-B (Amp) was used as a drug control. The experiment was performed in triplicate sets. The average followed by different letters are statistically different (p < 0.05).

*Cytochrome c* is a vital electron transport chain component loosely attached to the inner part of the mitochondrial membrane. The discharge of *cytochrome c* from mitochondria to the cytosol leads to the triggering of caspases, a crucial factor that triggers apoptotic cell death (Liao and Sun, 2018). Measuring the quantity of *cytochrome c* leaked from the mitochondria to the cytosol or from the cells to the culture medium is a potent tool for inspecting apoptotic cell death. To verify whether the EECS triggers the *cytochrome c* release from mitochondria to the cytosol, the changes in the *cytochrome c* level in both were measured. From the results, it was clear that there was a significant increase in the *cytochrome c* release from mitochondria to cytosol when the concentration of EECS increased. In the control (drug-free) group, the relative fluorescent value for mitochondrial and cytosolic *cytochrome c* was considered as 1.0. Significant *cytochrome c* release was observed in EECS-treated *Candida* cells at 2× MIC concentration, with relative fluorescent values of 0.84 and 1.37 for mitochondrial and cytosolic *cytochrome c*, respectively. Similarly, in the *C. albicans* treated with a MIC concentration of EECS, the relative fluorescent values for mitochondrial and cytosolic *cytochrome c* were 0.91 and 1.21, respectively. But in the case of lower concentration, i.e. 0.5× MIC the relative fluorescent value was found to be 0.97 and 1.11 for mitochondrial and cytosolic *cytochrome c*. Our study clearly showed a lower level of *cytochrome c* in the mitochondria when compared to the non-treated control samples (**Figure 9**). But, a higher *cytochrome c* level was observed in the cytosolic samples when compared to that of mitochondrial and control samples (**Figure 9**). These data clearly indicated the significant discharge of *cytochrome c* from mitochondria to the cytosol after EECS treatment.

### EECS significantly increases the Ca^2+^ levels in the cytosol and mitochondria

**Figure 10:**
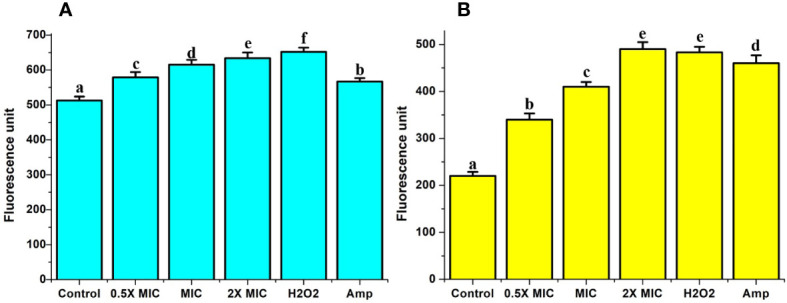
Analysis of cytosolic and mitochondrial Ca^2+^ level. **(A)** The relative fluorescence of cytosolic Ca^2+^ level by Fura-2AM analysis. **(B)** The relative fluorescence of mitochondrial Ca^2+^ level by Rhod-2AM analysis. The positive control used in the assay was H_2_O_2_ (2 mM), whereas amphotericin-B (Amp) was used as a drug control. The experiment was performed in triplicate sets. The average followed by different letters are statistically different (p < 0.05).

In addition to ROS, Ca^2+^ also displays a prominent role in the commencement and implementation of apoptosis in the organism (Jia et al., 2019). Enhanced Ca^2+^ levels in the cytosol cause permeabilization mitochondria and further release of proapoptotic factors, which eventually instigate the apoptotic pathway. In this investigation, we used Fluo-3 AM and Rhod-2 AM to study the level of cytosolic and mitochondrial Ca^2+^, respectively. The results from our study revealed significant increases in the intensity of fluorescence in both Fluo-3 AM and Rho-2 AM after treatment with different concentrations of EECS (**Figure 10**). The results obtained from our study pointed out that EECS caused a significant Ca^2+^ ion movement, which led to an increased accumulation of ions in the cytosol and mitochondria.

### EECS induced the activation of metacaspase

**Figure 11:**
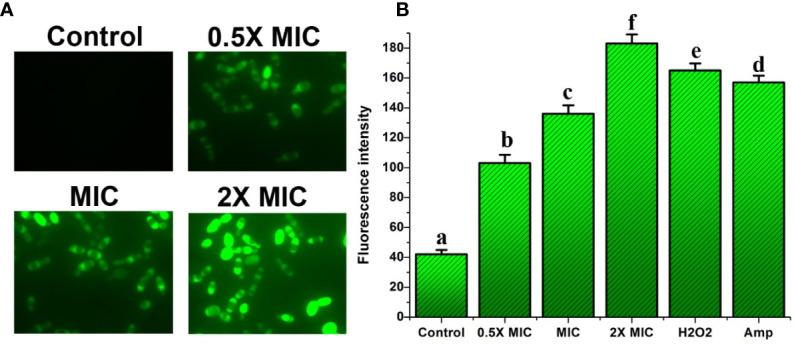
Analysis of caspase activation after treatment with EECS. **(A)** Metacaspase activity observed by FITC-VAD-FMK staining after the treatment with EECS. **(B)** Measurement of metacaspase triggering by FITC-VAD-FMK. The positive control used in the assay was H_2_O_2_ (2 mM), whereas amphotericin-B (Amp) was used as a drug control. The experiment was performed in triplicate sets. The average followed by different letters are statistically different (p < 0.05).

We investigated the activation of metacaspases during EECS-induced apoptosis using CaspACE FITC-VAD-FMK, a FITC-conjugated caspase inhibitor. The CaspACE FITCVAD-FMK can bind irretrievably to the activated caspase in apoptotic cells to exhale green fluorescence. The results of the present investigation recorded significant green fluorescence in EECS-treated *Candida* cells, demonstrating cleaved or activated metacaspases when compared to control cells (**Figure 11A**). As the EECS concentration increased from 0.5× MIC to 2× MIC, the green fluorescence intensity of *C. albicans* also increased, which pointed to the dose-dependent activation of metacaspases (**Figure 11B**). These results clearly demonstrated the role of metacaspase, a key regulator of apoptosis in *C. albicans* cells during EECS treatment.

### EECS significantly protected *G. mellonella* larvae from experimental candidiasis

**Figure 12:**
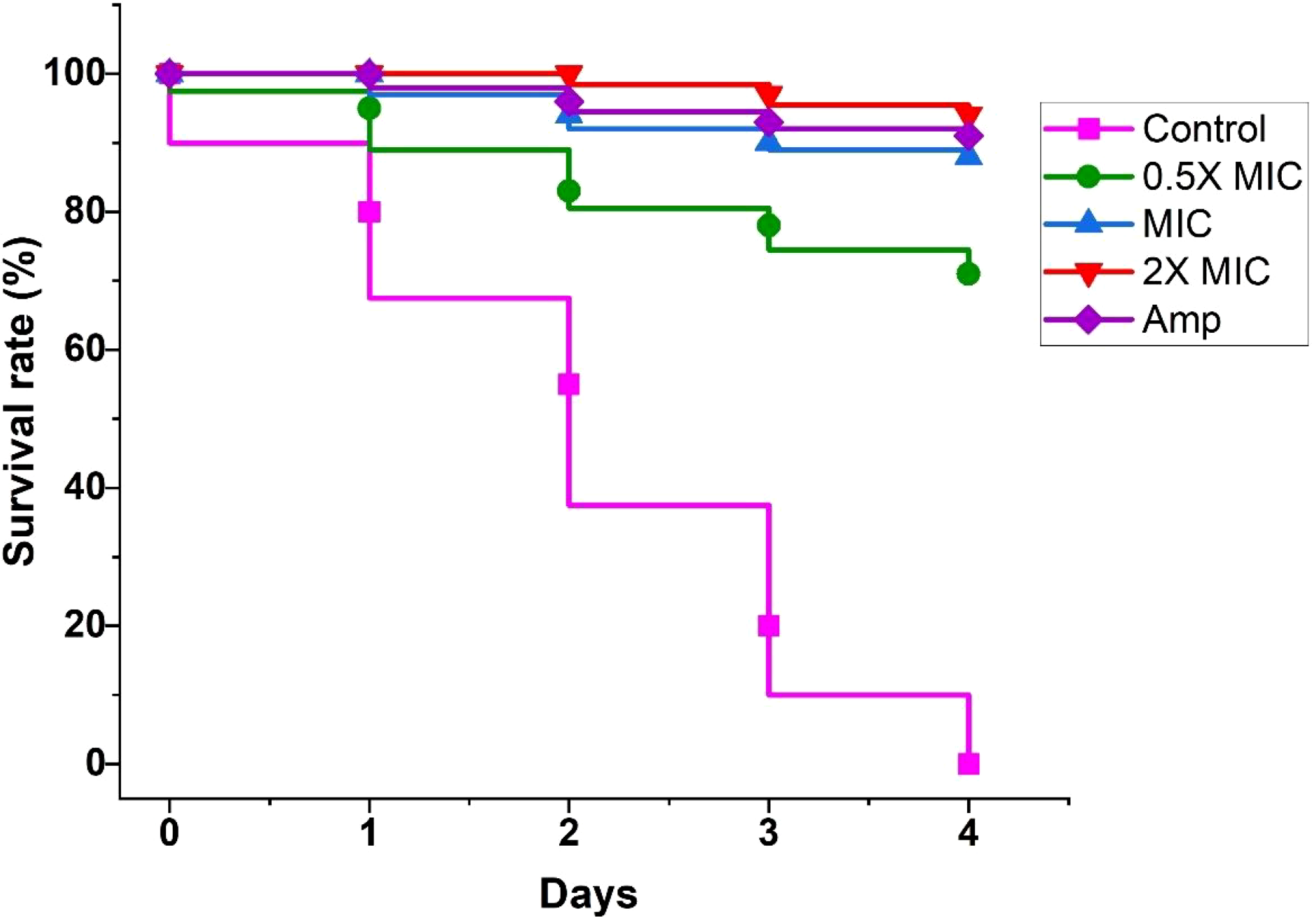
Survival curve of *G. mellonella* infected with *C. albicans* after treatment with EECS. Amphotericin-B (Amp) was used drug control. The outcome represented here came from the means of three replication.

*G. mellonella* larvae infected with *C. albicans* were employed in this study to assess the efficacy of EECS in the treatment of candidiasis *in vivo*. The survival assay was carried out mainly to evaluate the potency of EECS in *G. mellonella* larvae diseased with *C. albicans* (**Figure 12**). In the survival assay, *G. mellonella* larvae infected with *C. albicans* were incubated at 37°C for 4 days, and larval death was documented daily by inspecting visually. In this experiment, *C. albicans* infected larvae followed by EECS treatment significantly protected them from infection when related to the control sample (P < 0.05). Hence, from this study, it can be finalised that EECS significantly protects *G. mellonella* larvae from the mortality induced by *C. albicans*.

### Cytotoxicity

**Figure 13:**
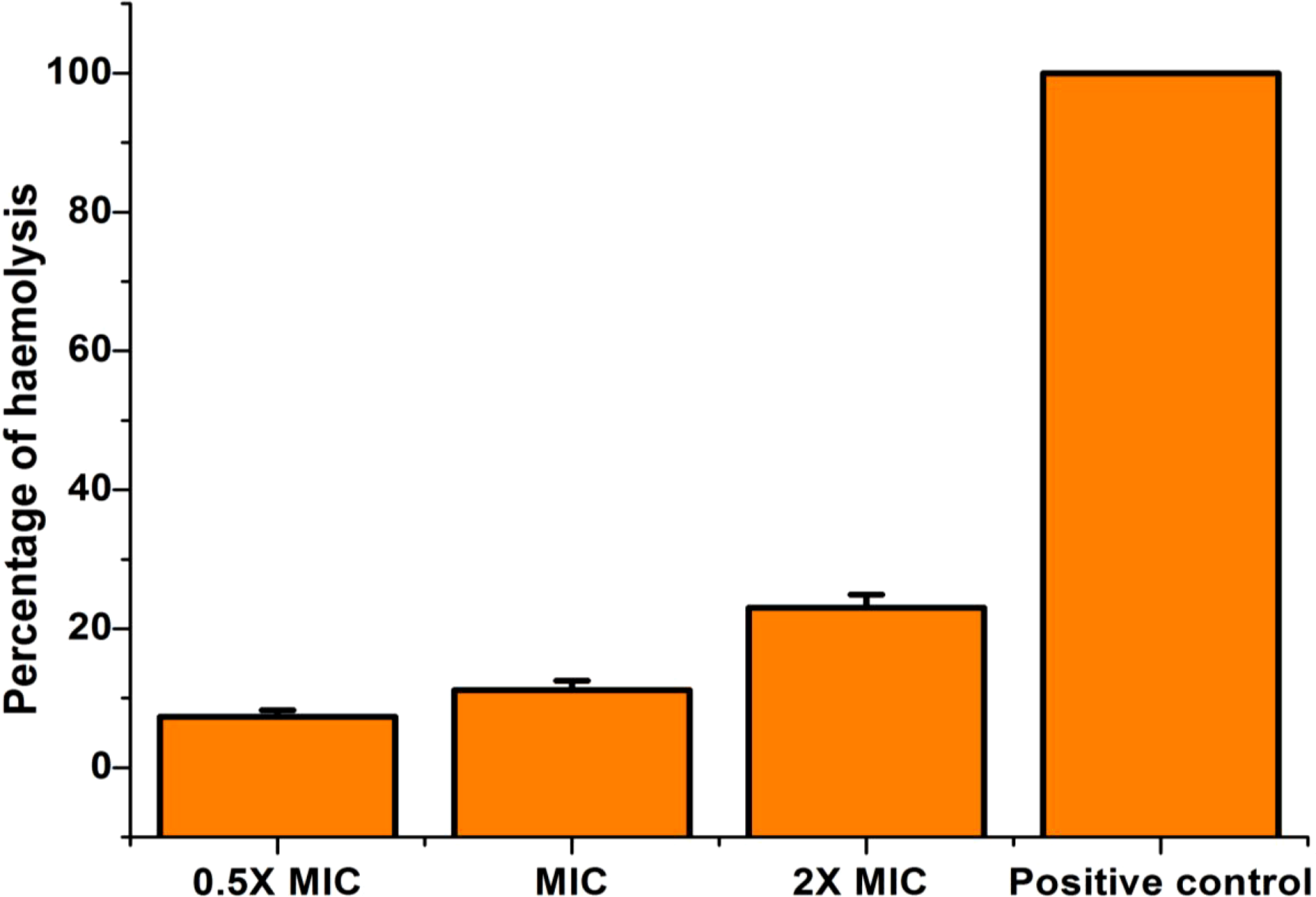
Cytotoxicity (hemolytic) activity of EECS in horse RBCs. EECS at various concentration was significantly less toxic when compared with positive control (which recorded 100% hemolysis). The negative control (PBS) recorded no hemolysis. The experiment was performed in triplicate sets.

From our study, it is clear that EECS has noteworthy anti-candidal activity and was able to activate apoptosis mediated cell death in *C. albicans*. Hence, the investigation of the toxicity level of this extract was pivotal. So, we conducted the cytotoxicity of EECS in horse RBCs. The percentage of hemolysis in the positive control was 100% while the negative control recorded no lysis. Compared to controls, the EECS at 2× MIC concentration recorded some hemolysis (**Figure 13**). Hemolysis displayed by the EECS was 10.75%, 13.49% and 26.38% for 0.5× MIC, MIC and 2× MIC concentrations, respectively. The data from this investigation clearly established that the EECS was significantly less toxic than the positive control, thus signifying its use for various in-vivo investigations in the future for the development of potent antifungal drugs.

## Discussion

At this moment, the growing number of various multidrug resistant (MDR) fungal pathogens, particularly *Candida* species, is reckoned as one of the world’s utmost serious public health issues, posturing challenges to the growth of the worldwide health system. Various *Candida* species are normal pathogenic yeast colonizers of humans, especially in the mouth, throat, gut, and vagina. *Candida* species can cause two major infections in humans: superficial and life-threatening systemic infections. The occurrence of infections associated with pathogenic *Candida* has increased worldwide, with fatality rates greater than 70% in specific patient populations. *C. albicans, C. tropicalis, C. glabrata, C. parapsilosis*, and *C. krusei* are the five major *Candida* species accountable for more than 90% of *Candida*-associated infections. Of these five species, *C. albicans* is responsible for causing significant infections. It is well reported that medicinal plants as well as phytochemicals are influential and affordable novel antifungal medications for the effective management of *Candida* infections with minimal or no side effects (Kumar et al., 2019). So, in the present investigation, we examined the anticandidal effects of the three extracts obtained from the seeds of *C. bonduc* against four major *Candida* species. Out of three extracts tested, EECS recorded significant effectiveness against *C. albicans*. Therefore, we endeavored to investigate the detailed study of the *C. bonduc* seed extract, especially on the mechanism of action of EECS in *C. albicans*.

*C*. *albicans *can easily colonize the tissues and implanted devices in the host easily and form biofilms, which are critical in pathogenesis and drug resistance. The conversion of yeast to hyphal cells is considered as a central role in the formation of biofilm and the associated pathogenesis of yeast infections (Sapaar et al., 2014). Numerous natural compounds, including phytochemicals, have been reported to hinder the biofilm* *developed by *albicans*, mainly by inhibiting the formation of hypha (Lee et al., 2019). The high fatality rates in *Candida-*associated infections are predominantly due to the inability of traditional remedies to kill multidrug-resistant cells embedded in biofilms (Nadine et al., 2019). This extracellular polymeric matrix provides physical protection to the Candida community, thus preventing the action of a wide range of commercially available antifungal medications on the market. Hence, searching for alternative antifungals that stop or destroy biofilm formation is absolutely necessary (Nadine et al., 2019). So, in this investigation, we have tested the effectiveness of the EECS in inhibiting the formation of biofilm by *C. albicans*. In our research, EECS significantly inhibited biofilm formation by the *C*. *albicans*. At a 2×MIC concentration, more than 95% of the biofilm was inhibited when compared with the control. Moreover, a significant reduction in the biofilm was also noticed at the MIC concentration of EECS. In this context, EECS could emerge as a possible candidate drug molecule against *C. albicans* in future, because it displays significant anticandidal activity besides exerting a substantial antibiofilm effect on *C*. *albicans *cells.

Numerous research studies suggest that phytochemicals can promote ROS production at high concentrations (Gaikwad and Srivastava, 2021; Li et al., 2022). It is well understood that ROS production is a universal mode of action of antifungal drugs, which contributes to their fungicidal activity against pathogenic organisms. For example, amphotericin-B exerts antifungal activity through the significant generation of ROS (Li et al., 2020). Many research studies in recent years have reported that ROS is a key regulator of apoptosis cell death in *Candida* (Tian et al., 2017b; Price et al., 2009). The production of ROS is one of the early factors associated with the initiation of the apoptotic pathway (Benaroudj et al., 2001). Interestingly, mitochondria exemplify the primary origin of intracellular ROS (Tian et al., 2017b). ROS generation and accumulation cause severe oxidative impairment to the various micro and macromolecules in cells, resulting in mitochondrial and DNA deterioration (Tian et al., 2017b). At the same time, ROS accumulation can induce metacaspase-dependent apoptosis in cells (Wu et al., 2010). Hence, we initially checked the ROS generation in *C. albicans *cells after being treated with various concentrations of EECS. A DCFH-DA assay was executed to examine the generation of intracellular ROS after drug treatment. This experiment showed a noteworthy increase in the generation of ROS in *C. albicans* after EECS treatment when compared to the untreated (control) sample. The generation of ROS by EECS might be connected with other apoptotic activity, especially caspase-mediated apoptosis. Moreover, it is further recited that ROS production can destruct the chromosomal DNA and speed up the decline of mitochondrial membrane potential (Tian et al., 2017b). All these were investigated later in the study.

The organism’s cell membrane acts as a barrier to guard cells from various external stresses, which is important for existence. As said earlier, various free radicals, especially ROS, can cause harm to the cell membrane due to their toxic action (Li et al., 2020). Moreover, excessive ROS generation can lead to the oxidation of various macromolecules and is also associated with severe cell membrane damage, which affects the membrane integrity of the cells (Kamat et al., 2000). Therefore, we checked the effectiveness of EECS in ROS-mediated cell membrane damage through the PI staining method. PI is a fluorescent dye that is highly cell membrane-impermeable and therefore cannot stain healthy cells. But it can quickly enter the damaged cell membrane and stain cells with bright red fluorescence by merging with DNA (Choi et al., 2012). To further confirm the damage of the membrane integrity, a PI staining experiment was conducted by exposing the *C. albicans* with various concentrations of EECS, followed by staining with PI. The PI will enter the *Candida* once the integrity of the membrane is damaged due to the effects of EECS. According to **Figure 5**, uptake of PI by the EECS-treated *C. albicans* cells increases when the dose increases. These results further indicated the enhanced membrane permeabilization and loss of membrane integrity due to the treatment of EECS.

Mitochondria are a vital organelle for energy metabolism, which is further linked to cellular physiology and integrity (Lee and Lee, 2017). Furthermore, they participate in ROS production and regulation, and when mitochondria fail, they can activate apoptotic mediated cell death *via* the generation of various apoptotic signals. The majority of phytochemicals that induce apoptosis in yeast involve the mitochondria-dependent pathway. As we know, the production of ROS triggers mitochondrial dysfunction (Choi and Lee, 2015), which leads to mitochondrial membrane depolarization in cells (Pereira et al., 2008). Furthermore, increased ROS generation causes severe damage to the components of the mitochondria, which in turn affects the membrane integrity. Therefore, we studied mitochondrial damage in *C. albicans* succeeding EECS treatment. Through this experiment, we established the existence of mitochondrial depolarization and the involvement of mitochondrial damage in EECS-treated *C. albicans* using Rh-123 analysis. Finally, from the results of Rh-123 staining, it was clear that ROS generation after EECS treatment is well associated with mitochondrial dysfunction in *C. albicans*.

*Cytochrome* c is a major pro-apoptotic factor, rigidly bound to the inner mitochondrial membrane. When the mitochondrial membrane of the cells loses biological functions due to integrity or depolarization which will lead to the release of *cytochrome* c from the mitochondria. The released *cytochrome* c excites apoptotic signals. When the mitochondria are damaged, they can be readily translocated from the mitochondria to the cytoplasm (Wani et al., 2021) and support the activation of metacaspases (Wu et al., 2010). As the EECS induced the dysfunction of mitochondria and enhanced the permeability of the mitochondrial membrane, we next examined the translocation of *cytochrome* c from the mitochondria to the cytosol. Here we observed an enhanced level of *cytochrome* c* *in the cytosol when compared to the control cells (**Figure 7**), whereas the mitochondrial *cytochrome* c level decreased drastically (**Figure 7**). The variation in the cytosol and mitochondrial *cytochrome* c levels clearly pointed to the translocation of *cytochrome c* from the mitochondria to the cytoplasm. Cho and Lee (2011) further suggest that EECS-induced apoptosis may be further enabled through the initiation of the release of pro-apoptotic molecules. In addition to this, translocation of *cytochrome* c to the cytosol is an important process in apoptotic-mediated cell death.

Mitochondria play a crucial role in activating apoptosis in *C. albicans* exposed to EECS. The Rh-123 and *cytochrome* c release results clearly showed that EECS induced significant mitochondrial dysfunction and enhanced *cytochrome* c release into the cytoplasm from the mitochondria. As a result, it can be concluded that EECS causes mitochondrial dysfunction through increased ROS production, which can lead to apoptosis-mediated cell death in *C. albicans*.

Various ions play a vital role in regulating manifold cellular functions and signaling. Hence, the ionic imbalance can cause cell death due to cellular damage. Cellular ionic homeostasis plays a critical role in controlling apoptotic processes in the cell. Numerous studies have revealed that calcium and potassium ions are significant apoptosis initiating ionic factors (Yun and Lee, 2016; Lee and Lee, 2018). Ca^2+^ is an important signaling ion in cells that controls various critical biological processes. Amongst the several cellular signaling systems, ROS predominantly interacts with the Ca^2+^ signaling pathway (Kwun et al., 2021). As previously stated, EECS induces an increase in intracellular ROS, which can also cause an increase in intracellular Ca^2+^ levels. Usually, Ca^2+^ concentrations in the cytosol are usually low in resting states. But, Ca^2+^ inrushes into the cytosol in response to numerous external stresses happening in the organism and thus increases cytosolic Ca^2+^ concentrations that may act as apoptotic stimuli. To check Ca^2+^ levels after EECS treatment, we used Fura-2AM and Rhod-2AM, which are extremely cell-permeable and highly selective for free mitochondrial* *and cytosolic Ca^2+^ ions, respectively. This study established an EECS-induced* *significant* *enhancement in the cytosolic Ca^2+^ ion levels when compared to mitochondrial. An increased level of Ca^2+^ ions impedes the respiration of mitochondria, resulting in the progression of mitochondrial membrane permeability. This in turn emits various proapoptotic proteins (Li et al., 2020). As previously stated, we found enhanced ROS production leads to mitochondrial dysfunction due to mitochondrial membrane permeability. In addition to this, the rise in mitochondrial calcium stimulation will also act as a significant source of ROS (Lee and Lee, 2017).

Chromosomal condensation, DNA fragmentation, and caspase activation are considered the universal hallmarks of apoptosis (Hao et al., 2013). In apoptotic cells, during chromosomal DNA degradation, the DNA is cleaved into oligonucleotides with the help of the enzyme DNase. PS is a negatively charged phospholipid found in the cell membranes of eukaryotic organisms. As we know, the PS is exposed to the cell surface during the early apoptotic stage (Tian et al., 2017a). As a result, translocation of PS is a prominent biochemical and physiological characteristic feature of apoptosis. Our study detected PS externalization by means of annexin V-FITC/PI double staining methods. Annexin V binds firmly when PS is externalized to the outer leaflet of the cytoplasmic membrane of apoptotic cells (Tian et al., 2017a). The double staining of the cells by annexin V-FITC and PI permits the differentiation between normal, apoptosis and late apoptotic/necrotic cells. In this staining method, normal cells will be annexin V-/PI-, whereas apoptotic cells will be annexin V+/PI- and late apoptotic/necrotic cells will be annexin V+/PI+ (Seong and Lee, 2018). The annexin V-FITC and PI analysis of *C. albicans* cells after treatment with EECS are shown in **Figure 6**, which clearly shows the features of early apoptosis, i.e., annexin V+/PI-.

Furthermore, we also confirmed the DNA condensation and fragmentation using DAPI and TUNEL staining assays. DAPI is a DNA-specific probe that forms a prominent fluorescent composite by linking to the minor groove of the A-T rich sequences and pacifies nuclear condensation and fragmentation. The TUNEL and DAPI assays were used to detect apoptotic DNA cleavage, a clear indication of DNA fragmentation and nuclear condensation, a prominent marker of late apoptosis (Yun et al., 2016). The TUNEL and DAPI stained cells recorded the DNA and nuclear condensation/fragmentation as green and blue fluorescent staining when viewed under a fluorescent microscope. As a result, we confirmed that EECS-treated *Candida* cells induce apoptosis, which leads to chromosomal condensation, DNA fragmentation and the exposure of PS in *C*. *albicans*. Thus, it can be concluded that EECS contributes to apoptosis in *C. albicans*.

Apoptotic cell death in yeast (Candida) occurs through two major pathways. The first one is caspase-dependent (Madeo et al., 2002), and the second is a caspase-independent pathway (Susin et al., 1999). Statistical analysis of the various research studies on yeast showed that nearly 40% of apoptosis in *Candida* species is *via* a caspase-dependent pathway (Chen et al., 2014). ROS, followed by mitochondrial dysfunction and calcium accumulation, is a significant factor that triggers yeast apoptotic cell death through the activation of metacaspase in *Candida*. As we know, the EECS leads to significant ROS production in *C. albicans*, so we aimed to study the activation of metacaspase to unravel the basic mechanism behind the EECS-induced apoptosis in *C. albicans*. Here we evaluated the activation of caspase in *C. albicans* with the aid of CaspACE FITC-VAD-FMK. FITC-VAD-FMK is a fluorescent dye that binds explicitly to the active site of caspase. In the EECS-treated *Candida* cells, activation of caspase was detected clearly as green fluorescence through a fluorescent microscope, while the control cells exhibited no fluorescence. This result shows that the EECS significantly promoted metacaspase activation in *C. albicans*, clearly demonstrating that the apoptosis in *C. albicans* is interceded by a caspase-dependent apoptotic pathway.

In recent years, insects have been commonly used to assess the virulence of pathogenic microorganisms and further evaluate the *in-vivo* efficacy and toxicity of antimicrobial drugs (Jemel et al., 2020). *The G. mellonella* infection model was a commonly used insect model for evaluating the in-vivo efficacy of the drug because of its cost effectiveness and ease of handling (Jemel et al., 2020). Thus, in this investigation, we used a *G. mellonella* infection model to assess the efficacy of EECS. Cotter et al. (2000) described *G. mellonella* as a mini-host to investigate the toxicity of drugs in various *Candida species*. In addition to this, the larvae of *G. mellonella* can be easily incubated at 37°CC, a vital factor that plays a crucial role in the virulence of *C. albicans* in *in vivo* conditions (Lv et al., 2020). In our study, EECS significantly enhanced the survival time after infecting *G. mellonella with C. albicans*. The EECS greatly improved the efficacy of anticandidal activity in *in vivo* conditions, which is consistent with the effects exhibited in *in vitro* conditions.

Toxicity probing is a vital step toward the development of an antimicrobial drug. In the development of our study, EECS recorded prominent anti-candidal activity by generating ROS, mitochondrial dysfunction, calcium accumulation and thereby inducing cellular apoptosis. So, the toxicity of EECS was assessed using the hemolytic activity of horse erythrocytes. Lysis of RBC was not detected in untreated control cells, whereas the positive control recorded 100% hemolysis. In contrast to controls, EECS at MIC and 0.5X MIC concentrations caused far less hemolysis. These results confirmed that EECS was not toxic even at the tested concentration. This result positively supports the use of EECS in animal experiments in the future. However, experiments on cell lines/animal models and humans should be investigated to confirm the safety/efficacy of using EECS in managing antifungal infections.

## Conclusion

**Figure 14:**
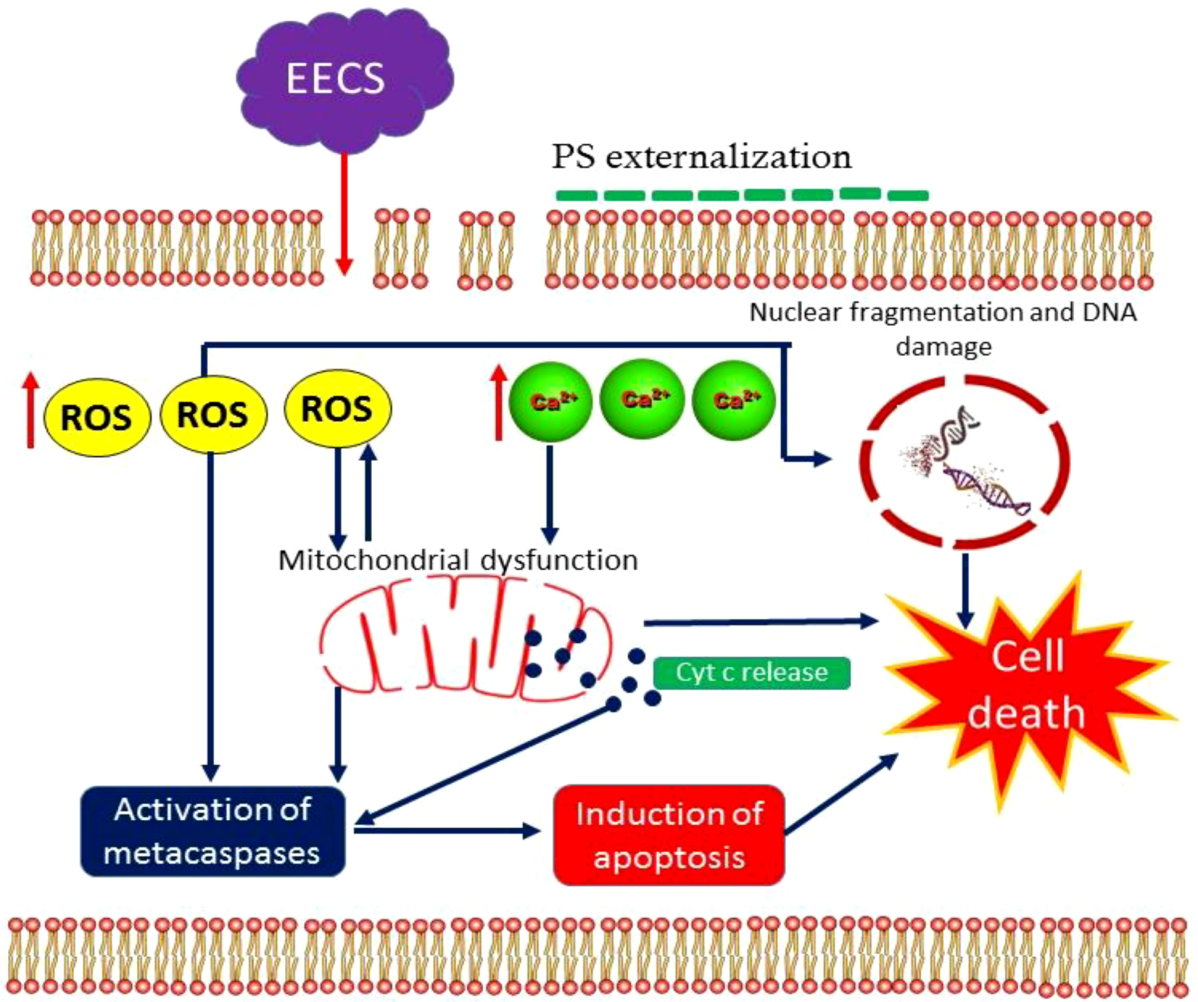
The action model of EECS in *C. albicans*.

To our best knowledge, this is the first report on the mode of action of an extract obtained from *C. bonduc* as an apoptosis inducer in *C. albicans* (**Figure 14**). Here we have tested the anticandidal activity of three extracts obtained from the *C. bonduc* seeds. Out of three seed extracts tested, ethanolic extract recorded a substantial effect on *C. albicans* and was selected for a detailed mechanism of action study. EECS causes excessive intracellular ROS generation, affecting the integrity of the cell membrane and upsurge intracellular Ca^2+^ levels in *C. albicans*. After that, a series of cellular events are initiated, which include mitochondrial membrane depolarization and homeostasis disruption of Ca^2+^ ions in the *C. albicans* cells. This represents the prevalence of mitochondrial dysfunction in *C. albicans*. Furthermore, typical hallmarks of apoptotic events were clearly detected after EECS treatment, including morphological changes associated with apoptosis, PS externalization, nuclear condensation and DNA fragmentation. Finally, EECS activates metacaspase-mediated cell death in *C. albicans*. Therefore, it can be concluded that EECS can trigger antifungal activity by activating apoptosis, which is mediated through a caspase-dependent pathway. The outcomes obtained from this investigation are of prodigious scientific implications in exposing the mechanism of antifungal activity of EECS, which may lay a strong platform for the pharmaceutical industry to develop this a novel drug. Our research team believes that the mechanism of EECS-induced yeast apoptosis may involve many more molecules and other more intricate events than previously thought. As we know, antifungal resistance signifies a foremost clinical challenge in treating various invasive fungal infections. In addition to this, these can exert serious adverse effects/toxicities that prevent their prolonged use. All these problems can be solved using phytochemicals, which are usually safe, effective, less toxic and cost-effective when compared to the antifungal medications presently available on the market. Hence, additional investigations are absolutely needed to illustrate the precise molecular mechanisms behind the apoptotic cell death in *C. albicans* induced by EECS. Thus, EECS holds the potential to be used as an antifungal medication for the management of various human pathogenic fungi, especially *C. albicans*, and further investigation of the therapeutic potential is warranted for the development of this medication.

## Data availability statement

The original contributions presented in the study are included in the article/supplementary material. Further inquiries can be directed to the corresponding author.

## Author contributions

SS and HN conceived and directed this research. SS and KN performed the experiment, analyzed the data and wrote the manuscript. KN processed the figures. SS and HN revised the manuscript. All the authors have read and approved the submitted manuscript version. All authors contributed to the article and approved the submitted version.

## Acknowledgments

Authors give adored Pranams to “Aadhyathma Chinthalayesan”, Chinthalaya Ashram, Pothencode, Trivandrum, Kerala, India for his benevolence and blessings. The authors would like to thank the Managing Director and Directors of Pankajakasthuri Herbals India Pvt. Ltd., Poovachal, Kattakada, Trivandrum, India for providing the essential infrastructure, amenities and financial support for this study.

## Conflict of interest

The authors declare that the research was conducted in the absence of any commercial or financial relationships that could be construed as a potential conflict of interest.

## Publisher’s note

All claims expressed in this article are solely those of the authors and do not necessarily represent those of their affiliated organizations, or those of the publisher, the editors and the reviewers. Any product that may be evaluated in this article, or claim that may be made by its manufacturer, is not guaranteed or endorsed by the publisher.
